# Study Protocol: Investigating the Effects of Transcranial Pulse Stimulation in Parkinson’s Disease

**DOI:** 10.3390/bioengineering12070773

**Published:** 2025-07-17

**Authors:** Anna Carolyna Gianlorenço, Lucas Camargo, Elayne Borges Fernandes, Elly Pichardo, Huan Jui Yeh, Dilana Hazer-Rau, Rafael Storz, Felipe Fregni

**Affiliations:** 1 Spaulding Neuromodulation Center and Center for Clinical Research Learning, Spaulding Rehabilitation Hospital, Harvard Medical School, Boston, MA 02115, USA; gianlorenco@ufscar.br (A.C.G.); lcamargo@mgh.harvard.edu (L.C.); elayne@estudante.ufscar.br (E.B.F.); epichardo@mgb.org (E.P.); yeti1102@hotmail.com (H.J.Y.); 2 Neurosciences Laboratory, Physical Therapy Department, Federal University of Sao Carlos, Sao Carlos 13565-905, Brazil; 3 Department of Physical Medicine and Rehabilitation, Taipei Hospital, Ministry of Health and Welfare, New Taipei City 242033, Taiwan; 4 Institute of Public Health, National Yang Ming Chiao Tung University, Taipei 112304, Taiwan; 5 STORZ MEDICAL AG, 8274 Tagerwilen, Switzerland; hazer.dilana@storzmedical.com (D.H.-R.); storz.rafael@storzmedical.com (R.S.)

**Keywords:** Parkinson’s disease, brain stimulation, transcranial pulse stimulation

## Abstract

Parkinson’s Disease (PD) is a progressive neurodegenerative disorder marked by motor and non-motor symptoms, including cognitive decline, mood disturbances, and sensory deficits. While dopaminergic treatments remain the gold standard, they present long-term side effects and limited impact on non-motor symptoms. Transcranial Pulse Stimulation (TPS) has emerged as a promising adjunct therapy in neurological and psychiatric conditions, but its effects in PD remain underexplored. This open-label, single-arm trial protocol involves 14 PD participants and outlines a personalized 12-session treatment approach combined with a homogeneously distributed TPS intervention among patients with PD. The approach addresses the subject’s most prominent symptoms, as identified through validated clinical assessments, encompassing domains related to both motor and non-motor symptoms. Over 2.5 months, besides the intervention sessions, the 14 participants will undergo an MRI brain scan, a baseline assessment, a post-treatment assessment, and a 1-month follow-up assessment. The study aims to determine whether personalized TPS is a feasible and safe intervention and whether it improves PD symptoms across multiple functional domains. This study represents the first structured attempt to evaluate a multimodal, personalized TPS intervention in patients with PD. It addresses gaps in current treatment approaches and may support the development of future strategies for integrated, symptom-targeted neuromodulation.

## 1. Introduction

Parkinson’s disease (PD) is the second most common neurodegenerative disease, affecting at least one million individuals in the United States, with annual costs estimated at approximately USD 52 billion (about USD 160 per person living in the US) [1,2,3]. Its physiopathology primarily includes loss of dopaminergic neurons in the nigrostriatal pathway, leading to reduced striatum dopamine levels [2]. PD is a complex, age-related, and progressive disorder characterized primarily by motor dysfunctions, such as resting tremors, bradykinesia, and rigidity. However, non-motor symptoms can also be present, including cognitive impairment, affective disorders, mood disturbances, and olfactory and gustatory dysfunction. All these motor and non-motor PD features associated with PD’s physiopathology may lead to mood, anxiety, and sleep disorders, which can exacerbate deficits in memory, attention, and thinking processes [4]. As a result, the combination of motor and cognitive impairments, along with behavioral disturbances, can lead to disability and a significant impact on social and occupational functioning, patients’ and families’ quality of life (QOL), and patients’ risk of morbidity and mortality [5,6].

The clinical manifestations of PD are highly heterogeneous, with patients exhibiting diverse combinations of symptoms and varying rates of disease progression. This heterogeneity has been increasingly studied through multimodal data analyses, which have identified distinct PD subtypes such as tremor-dominant, postural instability and gait difficulty (PIGD)-dominant, and rapid eye movement sleep behavior disorder (RBD)-related forms [7,8]. These subtypes differ not only in clinical progression but also in cognitive function, non-motor symptom profiles, and treatment response, highlighting the need for individualized intervention strategies. For instance, PIGD patients are more likely to develop autonomic dysfunction and sleep disturbances, whereas the RBD subtype is associated with a higher risk of cognitive decline. Additionally, recent machine learning and imaging-based studies have revealed interindividual anatomical brain differences in PD, which may underlie the divergent trajectories of motor and cognitive symptom progression [9]. Therefore, personalized symptom assessment and targeted treatment planning may enhance therapeutic outcomes and improve the quality of life for individuals living with PD.

Current therapies include pharmacological (e.g., levodopa), non-pharmacological interventions (e.g., physical therapy and speech and language therapy [SLT]), and surgical procedures (e.g., Deep Brain Stimulation [DBS]), and mainly focus on enhancing motor symptoms [10,11,12]. Dopaminergic drugs represent the gold-standard therapy for improving motor function and reducing freezing of gait episodes in PD patients [13,14]. However, prolonged use of dopaminergic drugs increases the risk of numerous side effects, including dyskinesia and behavioral dysfunction such as impulse control disorders and mood disorders [10,11]. Furthermore, dopaminergic drugs may have a long-term negative impact on patients’ cognitive status [15].

In recent years, the number of non-invasive brain stimulation (NIBS) devices for treating PD has increased significantly. These therapies are appealing as they are low-cost techniques and have an excellent safety profile, lacking side effects like those related to pharmacological or surgical methods [16,17]. The two most common non-invasive techniques, Transcranial Magnetic Stimulation (TMS) and Transcranial Direct Current Stimulation (tDCS), have shown promise in treating PD; however, recent studies with these types of NIBSs have focused primarily on PD’s non-motor symptoms or memory, and a few of them targeted in freezing of gait [17,18,19,20,21,22,23].

### 1.1. Transcranial Pulse Stimulation (TPS) in the Context of Neurological and Psychiatric Conditions

**Figure 1:**
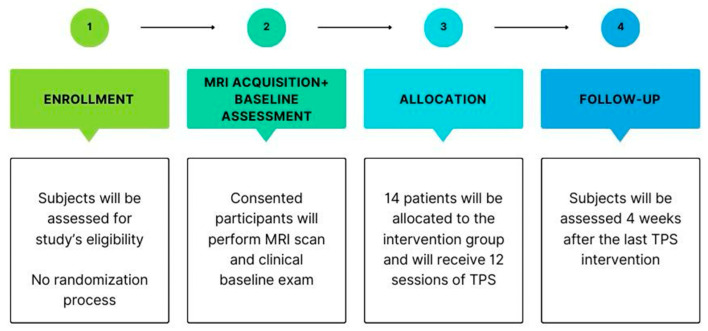
Study scheme of enrollment, intervention, and assessment timepoints along the study period. Follow-up will be performed 1 month after the last stimulation session.

TPS is a novel NIBS technology that applies repetitive, single high-pressure ultrashort shockwave pulses within the ultrasound frequency range to stimulate the brain. Since it uses a concomitant neuroimaging by neuro-navigation device (Figure 1), TPS enables more precise, focal, and deep brain modulation, reaching depths of up to 8 cm (about 3.15 in) into the brain, for instance, modulating subcortical areas, in a targeted manner, when compared with other NIBS [24,25].

Currently, TPS has been demonstrated to be a non-invasive and painless technique with no serious adverse effects, capable of modulating neural excitability and significantly improving many neurological conditions. The biological mechanisms hypothesis behind TPS therapy involves the modulation of vascular endothelial growth factor (VEGF) and brain-derived neurotrophic factor (BDNF), which have been associated with improved cerebral blood flow and angiogenesis, as well as effects on cell permeability, proliferation, and differentiation. These factors are believed to influence cell functions such as migration, proliferation, differentiation, and apoptosis, and may contribute to neuroplasticity and neuronal repair processes, as observed in preclinical studies [26,27]. Animal experiments have also shown that the blood–brain barrier (BBB) remains intact following TPS, supporting its safety profile [28].

Although it is a novel technology, TPS has shown many positive effects in other neurological disorders, such as mild neurocognitive disorders [29,30], Alzheimer’s disease (AD) [31,32,33,34,35,36,37,38], disorders of consciousness [39], autism spectrum disorder [40,41], attention deficit hyperactivity disorder [42,43], and depression [36,44]. In most of these studies, TPS has shown considerable potential in reducing depressive symptoms and enhancing different aspects of cognitive performance. A recent observational study reported that 10 sessions of TPS therapy were safe in PD subjects and led to improvements in clinical motor function in optimized PD patients with only mild and transient side effects (fatigue [50%], headache [30%], and dizziness [30%], which resolved within one day) [45]. Their findings showed a significant reduction in the Unified Parkinson’s Disease Rating Scale part III (UPDRS-III pre-TPS: 16.70 ± 8.85, UPDRS-III post-TPS: 12.95 ± 8.55; *p* < 0.001; Cohen’s d = 1.38), and none of the participants experienced worsening of their motor status. However, this study has some limitations, primarily related to its retrospective design, small sample size, lack of a sham control group, and the heterogeneity of the study population and protocol. A study showed positive effects on the motor functions of PD patients after even a single treatment with TPS of the motor cortex M1 in comparison to a sham group [46].

Findings from AD studies suggest that TPS might induce improvements in cognitive impairment and depression symptoms, with potential application in PD for both motor and non-motor symptoms [29,44]. Fong et al. (2023) [29] observed improved cognitive function in 19 elderly participants with mild neurocognitive disorder after six TPS open-label sessions. Results showed increasing MoCA scores: pre-TPS: (19.32 ± 4.52), post-TPS (21.16 ± 3.98), and 12-week follow-up (20.58 ± 4.29), with a one-way ANOVA effects of time of F (3, 54) = 4.99, *p* = 0.004. Moreover, Cheung et al. (2023) [44] in a pilot randomized controlled trial demonstrated a reduction in depression symptoms in the TPS group (mean difference = −6.60, *p* = 0.02) in comparison to the control group in a study with 30 subjects who underwent six sessions of TPS.

Therefore, TPS as an adjunct therapy for PD needs to be validated with further empirical support. In addition, the heterogeneity in the study populations and protocols within the PD-TPS domain poses a challenge in interpreting the results comprehensively [32,38].

### 1.2. The Present Study

This study protocol outlines for the first time a personalized treatment approach combined with a homogeneously distributed TPS intervention among patients with PD, tailored to address each individual’s most prominent symptoms as identified through validated clinical assessments, involving domains related to (i) motor symptoms, (ii) cognitive impairment, (iii) depression, (iv) sleep disorders, (v) fatigue, (vi) voice function, and (vii) loss of smell and taste. The primary hypothesis is that TPS, as an adjunct therapy, encourages plasticity-dependent changes that may improve PD’s overall symptoms. In addition to the seven core domains described here, this study also investigates the safety of TPS and its potential effects on improving the freezing of gait and overall QOL in patients with PD. This study aims to assess the safety, feasibility, and potential benefits of TPS in improving PD symptoms. We hypothesize that active TPS will be safe and feasible and might lead to significant improvements in PD symptoms, such as motor and cognitive function, depression, sleep disorders, fatigue, voice and speech function, taste and smell disorders, and QOL compared with the pre-intervention baseline measures. These preliminary findings can provide a foundation for designing future RCTs to validate the therapeutic efficacy of TPS.

## 2. Experimental Design

This is an open-label, single-arm pilot study (NCT06676995) designed to assess the safety and feasibility of TPS for improving overall PD symptoms, with a total sample size of 14 subjects to be conducted at Spaulding Rehabilitation Hospital, Cambridge, USA. This study consists of a consent and enrollment visit, a magnetic resonance imaging (MRI) brain scan, 12 sessions of TPS (3 sessions per week), and 6 assessment sessions—a baseline visit, after every 3 TPS sessions, and a 1-month follow-up visit after the last TPS session. Also, 1 assessment visit will be performed after every 3 TPS sessions. See the study scheme provided in Figure 1. Participants will primarily be recruited through extensive methods such as advertisements online and in local newspapers. The inclusion criteria are as follows: (i) age between 40 and 90 years old; (ii) diagnosis of “probable” or “possible” PD, as defined by the current clinical criteria [47] or as confirmed by a co-investigator neurologist or confirmation via medical records or a letter from a patient’s physician; (iii) disease stages 2 to 4 based on the UPDRS subdomain V (or Hoehn and Yahr scale); (iv) taking a stable regimen of anti-parkinsonian medications, defined as no changes in drug type, dosage, or administration frequency, for at least 30 days before study entry.

The exclusion criteria are as follows: (i) clinical features suggestive of other causes of parkinsonism/Parkinson’s plus syndromes; (ii) history of deep brain stimulation, brain ablation surgeries, or malignant mass brain lesions; (iii) history of schizophrenia, bipolar illness, or alcohol/drug abuse within the past six months; (iv) history of psychosis or suicidal thoughts; (v) contraindications to transcranial pulse stimulation (i.e., metal objects in the head, cortisone treatments within six weeks before the first stimulation session, CNS thrombosis); (vi) unstable metabolic and psychiatric conditions (e.g., uncontrolled diabetes, uncompensated cardiac issues, uncompensated pulmonary disease); (vii) contraindications to MRI according to MGB screening in the Martinos-Center (i.e., pacemaker, defibrillator, or wires other than sternal wires, metallic foreign body in the eye, or drug infusion devices—if the models of these devices are not compatible with MRI; (viii) pregnancy; (ix) epilepsy or disorders that significantly increase the likelihood of seizures, including: severe traumatic brain injury or congenital birth defects leading to seizures; (x) bed- or wheelchair-bound patients; (xi) non-English speakers, since speech will be assessed through the Consensus Auditory-Perceptual Evaluation of Voice (CAPE-V) questionnaire, which relies on voice deviance for natural conversational speech in English.

## 3. Materials and Equipment

### 3.1. MRI Data Acquisition

T1-weighted sequences are collected using a 3 T Siemens Skyra MRI scanner (Siemens Healthcare, Erlangen, Germany) equipped with a 20-channel head/neck coil. The sequence parameters include an in-plane resolution of 230 × 256 pixels, a maximum gradient of 40 mT/m, and a slice thickness of 1–2 mm, comprising 100 to 180 slice image, and covering the forehead to the occiput and from ear to ear.

### 3.2. TPS Protocol

Subjects will receive 12 TPS sessions, administered 3 times per week over 4 consecutive weeks, using a NEUROLITH^®^ device by STORZ MEDICAL AG, Tägerwilen, Switzerland. Each treatment session will involve the application of 10,000 pulses of focused shock waves. By default, 4000 shockwave pulses with an energy flux density of 0.25 mJ/mm^2^ will be distributed homogeneously across the brain areas (as standard intervention). A total of 2000 shockwave pulses will be applied to the foot soles. Previous reports regarding patients receiving foot sole stimulation have highlighted the benefits of including the plantar surface in treatment protocols [48,49].

Clinical experience suggests that applying Extracorporeal Shock Wave Therapy (ESWT) to the plantar surface by using approximately 1500 impulses at an energy flux density between 0.10 and 0.15 mJ/mm^2^, improves the patients’ sense of ground contact and leads to a reduction in muscular stiffness in the lower limbs These effects contribute to improved mobility, gait initiation, lower limb muscle stiffness, reducing freezing episodes and overall comfort in PD [50].

The remaining 4000 shockwave pulses with an energy flux density of 0.25 mJ/mm^2^, as an individual intervention, will be distributed to up to four different brain areas based on each subject’s most prominent symptoms (Table 1). The application will be guided by a neuronavigational system using previously acquired magnetic resonance images, allowing real-time tracking of the standard and individualized target areas for precise targeting and even distribution of pulses.

## 4. Detailed Procedure

### 4.1. Intervention

The TPS session sequence will be (1) individual, (2) standard (homogeneously across all brain areas), and (3) foot sole. As previously noted, up to four specific and personalized brain regions of interest will be targeted for individual intervention. For all patients, one of these four areas will be the entire strip of the primary motor cortex (1000 pulses), and the other 3000 pulses will be chosen according to the patient’s three most important clinical features.

**Figure 2:**
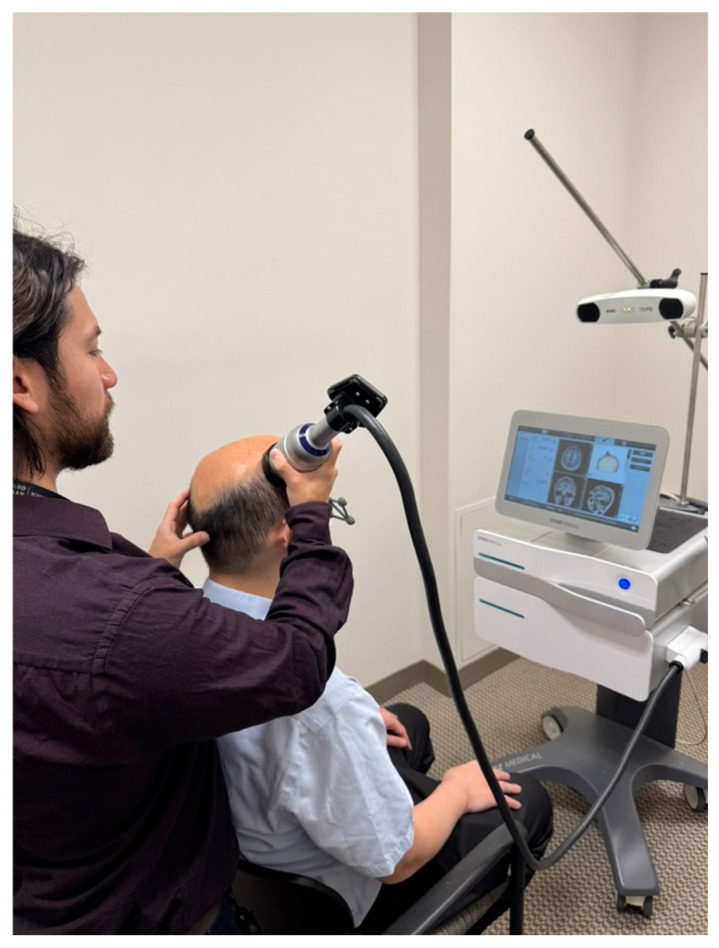
Study setup for the treatment of a patient with TPS using 3D navigation for personalized therapies.

During treatment, a camera system will monitor the positions of both the handpiece and the patient’s head using goggles equipped with infrared markers. This individual real-time tracking system allows for standardized focal brain stimulation across the entire study population, ensuring proper movement of the handpiece over the skull, as displayed in Figure 2. Furthermore, it will enable the tracking of the stimulation pulses, with each pulse leaving a colored mark in the visualization. The TPS intervention will use an energy flux density of 0.25 mJ/mm^2^ at a focus depth of 5 cm, with the useful energy dispersed between 2 and 8 cm. The frequency of application (pulse repetition rate) will be set at 4 Hz, with a coupling medium of water-based gel applied to the scalp.

**Figure 3:**
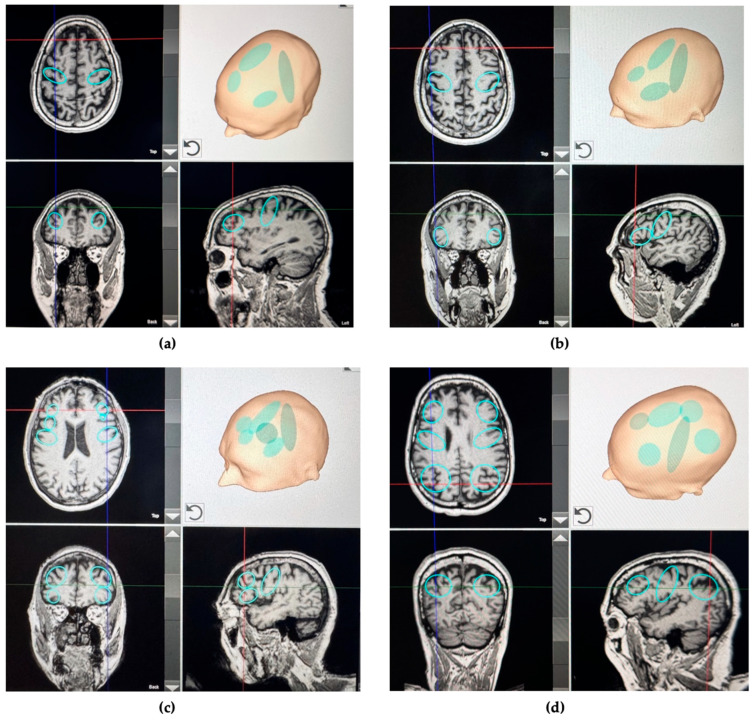
Examples of the TPS intervention strategy based on baseline assessments: (**a**) UPDRS-III score > 33 and a BDI score ≥ 14, pulse distribution: 1000 on M1 (each hemisphere), 1000 DLPFC (each hemisphere), and 4000 distributed across the brain areas (as standard intervention); (**b**) UPDRS-III score > 33 and VHI-10 ≥ 11, pulse distribution: 1000 on M1 (each hemisphere) and 1000 inferior frontal cortex extending on the left to Broca’s area (each hemisphere); (**c**) SCOPA-COG ≤ 24 and VHI-10 ≥ 11, pulse distribution: 500 on M1 (each hemisphere), 750 DLPFC (each hemisphere), and 750 inferior frontal cortex extending on the left to Broca’s area (each hemisphere); (**d**) PFS-16 binary score of >8, PDSS-2 score ≥ 18, 500 on M1 (each hemisphere), 750 DLPFC (each hemisphere), and 750 PPC. Additionally, 4000 will be distributed across the brain areas as a standard intervention. Horizontal and vertical lines reflect the position of the Coronal, Sagittal and Axial planes. Circular and oval shapes mark the areas of interest for the intervention.

During TPS, a generous amount of bubble-free ultrasound gel is applied sequentially to the skin and hair in the treatment area to prevent any acoustic impedance barriers. TPS application will be conducted in a continuous sweeping motion, lasting approximately 40 to 45 min per session, resulting in a total applied energy of 50 Joules (5 mJ/pulse with 0.25 mJ/mm^2^). The handpiece will be kept perpendicular to the skull for highly focused applications throughout the treatment. The entire treatment session will then be recorded for subsequent evaluation of the precise localization of individual intracerebral pulses, as presented in Figure 3.

The 12-session protocol consisting of 40 to 45 min of treatment over 4 weeks for PD, was developed, suggesting that frequent and consistent transcranial pulsed stimulation (TPS) sessions can maximize neuroplasticity and symptom management benefits. Additionally, this protocol is supported by prior studies demonstrating that repeated sessions over a short duration may optimize therapeutic outcomes by maintaining the neural changes induced by TPS, and that administering the treatment over a 3–4 week period may enhance both the efficacy and safety of TPS [31,34,36]. In contrast to other neuromodulation techniques such as tDCS and TMS, TPS has the potential to be applied less often, with more focused areas being treated.

### 4.2. Assessments and Outcomes

To assess motor and non-motor functions in PD subjects, this study protocol proposes the use of the following assessments: Unified Parkinson’s Disease Rating Scale (UPDRS) [51], Non-Motor Symptoms Scale (NMSS) [52], SCales for Outcomes in PArkinson’s disease-COGnition (SCOPA-COG) scale [53], Freezing of Gait Questionnaire (FOGQ) [54], Timed Up and Go (TUG) test [55], Parkinson’s Disease Sleep Scale (PDSS-2) [56], Parkinson’s Disease Fatigue Scale (PFS-16) [57], Beck Depression Inventory-Second Edition (BDI-II) [58], Visual Analog Mood Scale (VAMS) [59], Visual Analog Scale (VAS) for Olfactory and Gustatorial evaluation [60], Voice Handicap Index (VHI-10) [61], Consensus Auditory-Perceptual Evaluation of Voice (CAPE-V) [62], Voice acoustic characteristic [63], Parkinson’s Disease Questionnaire-39 (PDQ-39) [64], VAS for Pain; Brief Neurological Exam; Acceptability of Intervention Measure (AIM) scale and Feasibility of Intervention Measures (FIM) scale [65], and Patient’s Qualitative Assessment of Treatment—Real-World (PQAT-RW) [66]. The intervention strategy is defined after the baseline visit (visit 3), based on the pre-defined criteria displayed in Table 1 and Figure 3.

### 4.3. Safety

Adverse effects (AEs) questionnaires will be applied after every intervention session, allowing participants to grade the severity (mild, moderate, or severe) and indicate the perceived relationship to the intervention (none, remote, possible, probable, or related). In addition, a 20-min resting-state EEG will be conducted to monitor brain activity and investigate potential epileptiform discharges at baseline, after the first session, and after every three sessions (weekly), including a follow-up. Although not intended as a sole safety measure, EEG data will complement our clinical monitoring. Safety will also be assessed through clinical outcome scales (UPDRS, NMSS, and SPMSQ), where the absence of significant deterioration between baseline and post-intervention will support safety conclusions.

### 4.4. Data and Statistical Analysis

Data forms and questionnaires will be coded in a standardized format and entered into our database. Digital measures/recordings will be similarly tracked in our database and regularly backed up. Analyses will be conducted using standard statistical software such as STATA version 18.5 and R version 4.4.2.

The primary outcome will be a change in the UPDRS total score from the baseline. Secondary outcomes include change scales that measure improvements in UPDRS subdomains, motor and cognitive function, mood symptoms, sleep disorder, fatigue, voice quality, olfactory and gustatory disorder, freezing of gait episodes, and QOL comparing pre- and post-TPS. These results will be used to determine the best effect of TPS intervention for a more extensive study in patients with PD. Differences between the two groups (pre- and post-TPS) will be tested using a paired Student’s *t*-test, and, in addition, we will also test the time effect in general mixed regression models adjusting for age, gender, and PD severity. This model will be used for the primary and secondary outcomes. Secondary analyses will be conducted as exploratory, and for this reason, we will not apply formal correction for multiple comparisons. Since this is a pilot study with a small sample size, our aim is to identify possible trends and generate hypotheses for future confirmatory trials. Accordingly, all results from secondary analyses will be interpreted with caution. A similar analysis will be conducted for the adverse effects. We will also use the uncorrected *p*-value for safety analysis to increase the likelihood of detecting harmful adverse effects.

### 4.5. Sample Size and Power Calculation

To determine the sample size for this study, we considered the change in patients’ overall symptoms measured via the UPDRS from baseline as the primary outcome for our statistical analysis. Based on our pilot study of the effects of TPS in another neurodegenerative condition, we used the smallest effect size of d = 1.5 [31]. We calculated a minimum sample size of 14 participants for a within-group two-tailed paired *t*-test. Although a sample size of 14 subjects is relatively small, it can still provide valuable preliminary data and insights into the feasibility and potential efficacy of the intervention. This pilot study focuses on exploring trends, feasibility, and potential effect sizes rather than achieving statistical significance. However, a sample size of 14 subjects would provide a power of at least 80% (alpha = 0.05) to detect a mean difference of greater than 30% in overall symptom improvement between the pre- and post-stimulation, which is also what our AD study has shown [31]. This AD pilot study with only 10 patients could demonstrate a significant reduction in neuropsychiatric symptoms with large effect sizes (d = 1.5 and d = 2.3). Note, data from trials using MDS-UPDRS when investigating other NIBS therapies (tDCS and TMS) in PD patients was used to estimate the variances for this power analysis, which showed around 20% standard deviation and an effect size of about d = 1.29, even though the studies analyzed other NIBS rather than TPS [67,68]. Although it could interfere with the study power, we do not account for potential dropouts to calculate our sample size due to the preliminary analysis and small sample size. For this reason, the results from our pilot study will inform the design of future larger-scale studies and contribute to developing hypotheses for further investigation.

## 5. Expected Results

The proposed study aims to evaluate the safety and efficacy of TPS in addressing both motor and non-motor symptoms of PD, utilizing a sophisticated neuronavigational system to personalize the intervention based on individual patient symptoms and requirements. Despite the inherent limitations associated with this pilot open-label trial—such as a small sample size, absence of a control group, and potential placebo effects—this research can generate critical preliminary data to inform future randomized, double-blinded, controlled studies. We expect to observe significant improvements in motor and non-motor symptoms after 12 sessions of TPS, with remaining effects after a 1-month follow-up. Additionally, the reason for assessing subjects after every three intervention sessions is that we might observe improvements in symptoms such as tremor and loss of smell and taste in less than 12 TPS sessions. Moreover, as TPS, a non-invasive neuromodulation method, is known for its minor side effects, typically limited to mild local discomfort or pressure, we do not expect to observe severe adverse events, proving that TPS is a safe technique for this population.

This study protocol distinguishes itself from other neuromodulation studies through TPS’s capability for individualized, precise, focused brain stimulation that is trackable in real time throughout the intervention. Furthermore, TPS allows pulses to be strategically directed to different brain regions and specific target areas of interest. An additional strength of this protocol is its structured assessment schedule after every three intervention sessions, allowing investigators to evaluate the minimal number of sessions required to achieve significant improvements in patients with PD. It is important to highlight that the TPS equipment can deliver sham stimulation, which maintains the same features as active stimulation but is indistinguishable from active stimulation and replicates all operational aspects such as machine sounds and procedural setup, thereby facilitating blinded controlled trials in the future. In the long term, if TPS proves its safety and efficacy in the PD population, this non-invasive neuromodulation technique can be applied in clinical practice as a targeted-individualized treatment to improve the quality of life for patients living with PD.

## Figures and Tables

**Table 1:** Representation of the patients’ symptoms and target brain regions of interest for TPS individualized intervention.

Symptoms	Clinical Feature	Test Definition	Brain Region of Interest
Motor impairment	Moderate to severe rigidity, freezing of gait, postural instability, tremor, and bradykinesia	UPDRS-III score > 33	Bilateral and entire strip of the primary motor cortex (M1)
Cognitive impairment	Mild memory and executive function alterations	SCOPA-COG ≤ 24	Bilateral dorsolateral prefrontal cortex (DLPFC)
Psychiatric disorder	Depression	BDI-II score ≥ 14	Bilateral dorsolateral prefrontal cortex (DLPFC)
Sleep disorder	Moderate to severe sleep disturbance	PDSS-2 score ≥ 18	Bilateral posterior parietal cortex (PPC)
Fatigue	Moderate to severe fatigue	PFS-16 binary score of >8	Bilateral dorsolateral prefrontal cortex (DLPFC)
Voice/speech dysfunction	Moderate to severe dysarthria and poor voice control	VHI-10 score ≥ 11	Bilateral inferior frontal cortex extending on the left to Broca’s area
Taste and smell disorders	Moderate or severe olfactory and gustatory dysfunction	VAS for taste or smell ≥ 4	Bilateral orbitofrontal

## Data Availability

Not applicable.

## Abbreviations

The following abbreviations are used in this manuscript:

AD	Alzheimer’s Disease
AE	Adverse Effects
AIM	Acceptability of Intervention Measure
BBB	Blood–Brain Barrier
BDI	Beck Depression Inventory
BDNF	Brain-Derived Neurotrophic Factors
CAPE-V	Consensus Auditory-Perceptual Evaluation of Voice
DBS	Deep Brain Stimulation
DLPFC	Dorsolateral Prefrontal Cortex
EEG	Electroencephalogram
ESWT	Extracorporeal Shock Wave Therapy
FIM	Feasibility of Intervention Measures
FOGQ	Freezing of Gait Questionnaire
M1	Motor Cortex
MRI	Magnetic Resonance Imaging
NIBS	Non-Invasive Brain Stimulation
NMSS	Non-Motor Symptoms Scale
PD	Parkinson’s Disease
PDQ-39	Parkinson’s Disease Questionnaire-39
PDSS-2	Parkinson’s Disease Sleep Scale
PFS-16	Parkinson’s Disease Fatigue Scale
PIGD	Postural Instability And Gait Difficulty
PPC	Posterior Parietal Cortex
PQAT-RW	Patient’s Qualitative Assessment of Treatment–Real-World
QOL	Quality Of Life
RBD	Rapid Eye Movement Sleep Behavior Disorder
SCOPA-COG	SCales for Outcomes in PArkinson’s disease-COGnition
SLT	Speech and Language Therapy
tDCS	Transcranial Direct Current Stimulation
TMS	Transcranial Magnetic Stimulation
TPS	Transcranial Pulse Stimulation
TUG	Timed Up and Go
UPDRS	Unified Parkinson’s Disease Rating Scale
VAMS	Visual Analog Mood Scale
VAS	Visual Analog Scale
VEGF	Vascular Growth Factors
VHI-10	Voice Handicap Index

## References

[1] Grotewold N., Albin R.L. (2024). Update: Descriptive epidemiology of Parkinson disease. Park. Relat. Disord..

[2] Simon D.K., Tanner C.M., Brundin P. (2020). Parkinson Disease Epidemiology, Pathology, Genetics, and Pathophysiology. Clin. Geriatr. Med..

[3] De Lau L.M., Breteler M.M. (2006). Epidemiology of Parkinson’s disease. Lancet Neurol..

[4] Marsh L. (2013). Depression and Parkinson’s disease: Current knowledge. Curr. Neurol. Neurosci. Rep..

[5] Berganzo K., Tijero B., Gonzalez-Eizaguirre A., Somme J., Lezcano E., Gabilondo I., Fernandez M., Zarranz J.J., Gomez-Esteban J.C. (2016). Motor and non-motor symptoms of Parkinson’s disease and their impact on quality of life and on different clinical subgroups. Neurologia.

[6] Rahman S., Griffin H.J., Quinn N.P., Jahanshahi M. (2008). Quality of life in Parkinson’s disease: The relative importance of the symptoms. Mov. Disord..

[7] Fereshtehnejad S.M., Zeighami Y., Dagher A., Postuma R.B. (2017). Clinical criteria for subtyping Parkinson’s disease: Biomarkers and longitudinal progression. Brain.

[8] Deng X., Mehta A., Xiao B., Ray Chaudhuri K., Tan E.K., Tan L.C. (2025). Parkinson’s disease subtypes: Approaches and clinical implications. Park. Relat. Disord..

[9] Hahnel T., Raschka T., Sapienza S., Klucken J., Glaab E., Corvol J.C., Falkenburger B.H., Frohlich H. (2024). Progression subtypes in Parkinson’s disease identified by a data-driven multi cohort analysis. npj Park. Dis..

[10] Saba R.A., Maia D.P., Cardoso F.E.C., Borges V., LA F.A., Ferraz H.B., Barbosa E.R., Rieder C.R.M., da Silva D.J., Chien H.F. (2022). Guidelines for Parkinson’s disease treatment: Consensus from the Movement Disorders Scientific Department of the Brazilian Academy of Neurology—Motor symptoms. Arq. Neuropsiquiatr..

[11] Church F.C. (2021). Treatment Options for Motor and Non-Motor Symptoms of Parkinson’s Disease. Biomolecules.

[12] Scelzo E., Beghi E., Rosa M., Angrisano S., Antonini A., Bagella C., Bianchi E., Caputo E., Lena F., Lopiano L. (2019). Deep brain stimulation in Parkinson’s disease: A multicentric, long-term, observational pilot study. J. Neurol. Sci..

[13] Murakami H., Shiraishi T., Umehara T., Omoto S., Iguchi Y. (2023). Recent Advances in Drug Therapy for Parkinson’s Disease. Intern. Med..

[14] Gao C., Liu J., Tan Y., Chen S. (2020). Freezing of gait in Parkinson’s disease: Pathophysiology, risk factors and treatments. Transl. Neurodegener..

[15] Poletti M., Bonuccelli U. (2013). Acute and chronic cognitive effects of levodopa and dopamine agonists on patients with Parkinson’s disease: A review. Ther. Adv. Psychopharmacol..

[16] Wagner T., Valero-Cabre A., Pascual-Leone A. (2007). Noninvasive human brain stimulation. Annu. Rev. Biomed. Eng..

[17] Fregni F., Simon D.K., Wu A., Pascual-Leone A. (2005). Non-invasive brain stimulation for Parkinson’s disease: A systematic review and meta-analysis of the literature. J. Neurol. Neurosurg. Psychiatry.

[18] Wu A.D., Fregni F., Simon D.K., Deblieck C., Pascual-Leone A. (2008). Noninvasive brain stimulation for Parkinson’s disease and dystonia. Neurotherapeutics.

[19] Boggio P.S., Ferrucci R., Rigonatti S.P., Covre P., Nitsche M., Pascual-Leone A., Fregni F. (2006). Effects of transcranial direct current stimulation on working memory in patients with Parkinson’s disease. J. Neurol. Sci..

[20] Cantello R., Tarletti R., Civardi C. (2002). Transcranial magnetic stimulation and Parkinson’s disease. Brain Res. Rev..

[21] Dong K., Zhu X., Xiao W., Gan C., Luo Y., Jiang M., Liu H., Chen X. (2022). Comparative efficacy of transcranial magnetic stimulation on different targets in Parkinson’s disease: A Bayesian network meta-analysis. Front. Aging Neurosci..

[22] Mi T.M., Garg S., Ba F., Liu A.P., Wu T., Gao L.L., Dan X.J., Chan P., McKeown M.J. (2019). High-frequency rTMS over the supplementary motor area improves freezing of gait in Parkinson’s disease: A randomized controlled trial. Park. Relat. Disord..

[23] Valentino F., Cosentino G., Brighina F., Pozzi N.G., Sandrini G., Fierro B., Savettieri G., D’Amelio M., Pacchetti C. (2014). Transcranial direct current stimulation for treatment of freezing of gait: A cross-over study. Mov. Disord..

[24] Matt E., Kaindl L., Tenk S., Egger A., Kolarova T., Karahasanovic N., Amini A., Arslan A., Saricicek K., Weber A. (2022). First evidence of long-term effects of transcranial pulse stimulation (TPS) on the human brain. J. Transl. Med..

[25] Beisteiner R., Lozano A.M. (2020). Transcranial Ultrasound Innovations Ready for Broad Clinical Application. Adv. Sci..

[26] Wang B., Ning H., Reed-Maldonado A.B., Zhou J., Ruan Y., Zhou T., Wang H.S., Oh B.S., Banie L., Lin G. (2017). Low-Intensity Extracorporeal Shock Wave Therapy Enhances Brain-Derived Neurotrophic Factor Expression through PERK/ATF4 Signaling Pathway. Int. J. Mol. Sci..

[27] Zhang J., Kang N., Yu X., Ma Y., Pang X. (2017). Radial Extracorporeal Shock Wave Therapy Enhances the Proliferation and Differentiation of Neural Stem Cells by Notch, PI3K/AKT, and Wnt/beta-catenin Signaling. Sci. Rep..

[28] Karakatsani M.E., Nozdriukhin D., Tiemann S., Yoshihara H.A.I., Storz R., Belau M., Ni R., Razansky D., Dean-Ben X.L. (2025). Multimodal imaging of murine cerebrovascular dynamics induced by transcranial pulse stimulation. Alzheimer’s Dement..

[29] Fong T.K.H., Cheung T., Ngan S.T.J., Tong K., Lui W.Y.V., Chan W.C., Wong C.S.M., Cheng C.P.W. (2023). Transcranial pulse stimulation in the treatment of mild neurocognitive disorders. Ann. Clin. Transl. Neurol..

[30] Lo H.K., Fong T.K., Cheung T., Ngan S.J., Lui W.V., Chan W.C., Wong C.S., Wong T.K., Cheng C.P. (2024). Enhanced Cognition and Modulation of Brain Connectivity in Mild Neurocognitive Disorder: The Promise of Transcranial Pulse Stimulation. Biomedicines.

[31] Shinzato G.T., Assone T., Sandler P.C., Pacheco-Barrios K., Fregni F., Radanovic M., Forlenza O.V., Battistella L.R. (2024). Non-invasive sound wave brain stimulation with Transcranial Pulse Stimulation (TPS) improves neuropsychiatric symptoms in Alzheimer’s disease. Brain Stimul..

[32] Cont C., Stute N., Galli A., Schulte C., Logmin K., Trenado C., Wojtecki L. (2022). Retrospective real-world pilot data on transcranial pulse stimulation in mild to severe Alzheimer’s patients. Front. Neurol..

[33] Beisteiner R., Hallett M., Lozano A.M. (2023). Ultrasound Neuromodulation as a New Brain Therapy. Adv. Sci..

[34] Beisteiner R., Matt E., Fan C., Baldysiak H., Schonfeld M., Philippi Novak T., Amini A., Aslan T., Reinecke R., Lehrner J. (2020). Transcranial Pulse Stimulation with Ultrasound in Alzheimer’s Disease-A New Navigated Focal Brain Therapy. Adv. Sci..

[35] Dorl G., Matt E., Beisteiner R. (2022). Functional Specificity of TPS Brain Stimulation Effects in Patients with Alzheimer’s Disease: A Follow-up fMRI Analysis. Neurol. Ther..

[36] Matt E., Dorl G., Beisteiner R. (2022). Transcranial pulse stimulation (TPS) improves depression in AD patients on state-of-the-art treatment. Alzheimer’s Dement..

[37] Popescu T., Pernet C., Beisteiner R. (2021). Transcranial ultrasound pulse stimulation reduces cortical atrophy in Alzheimer’s patients: A follow-up study. Alzheimer’s Dement..

[38] Chen X., You J., Ma H., Zhou M., Huang C. (2024). Transcranial pulse stimulation in Alzheimer’s disease. CNS Neurosci. Ther..

[39] Lohse-Busch H., Reime U., Falland R. (2014). Symptomatic treatment of unresponsive wakefulness syndrome with transcranially focused extracorporeal shock waves. NeuroRehabilitation.

[40] Cheung T., Ho Y.S., Fong K.H., Lam Y.T.J., Li M.H., Tse A.C., Li C.T., Cheng C.P., Beisteiner R. (2022). Evaluating the Safety and Efficacy of Transcranial Pulse Stimulation on Autism Spectrum Disorder: A Double-Blinded, Randomized, Sham-Controlled Trial Protocol. Int. J. Environ. Res. Public Health.

[41] Cheung T., Li T.M.H., Lam J.Y.T., Fong K.H., Chiu L.Y., Ho Y.S., Tse A.C., Li C.T., Cheng C.P., Beisteiner R. (2023). Effects of transcranial pulse stimulation on autism spectrum disorder: A double-blind, randomized, sham-controlled trial. Brain Commun..

[42] Cheung T., Yee B.K., Chau B., Lam J.Y.T., Fong K.H., Lo H., Li T.M.H., Li A.M., Sun L., Beisteiner R. (2024). Efficacy and safety of transcranial pulse stimulation in young adolescents with attention-deficit/hyperactivity disorder: A pilot, randomized, double-blind, sham-controlled trial. Front. Neurol..

[43] Cheung T., Chau B., Fong K.H., Lam J.Y.T., Lo H., Li M.H., Li A., Beisteiner R., Lei S., Yee B.K. (2023). Evaluating the efficacy and safety of transcranial pulse stimulation on adolescents with attention deficit hyperactivity disorder: Study protocol of a pilot randomized, double-blind, sham-controlled trial. Front. Neurol..

[44] Cheung T., Li T.M.H., Ho Y.S., Kranz G., Fong K.N.K., Leung S.F., Lam S.C., Yeung W.F., Lam J.Y.T., Fong K.H. (2023). Effects of Transcranial Pulse Stimulation (TPS) on Adults with Symptoms of Depression-A Pilot Randomized Controlled Trial. Int. J. Environ. Res. Public Health.

[45] Osou S., Radjenovic S., Bender L., Gaal M., Zettl A., Dorl G., Matt E., Beisteiner R. (2024). Novel ultrasound neuromodulation therapy with transcranial pulse stimulation (TPS) in Parkinson’s disease: A first retrospective analysis. J. Neurol..

[46] Manganotti P., Liccari M., Maria Isabella Lombardo T., Della Toffola J., Cenacchi V., Catalan M., Busan P. (2025). Effect of a single session of transcranial pulse stimulation (TPS) on resting tremor in patients with Parkinson’s disease. Brain Res..

[47] Postuma R.B., Poewe W., Litvan I., Lewis S., Lang A.E., Halliday G., Goetz C.G., Chan P., Slow E., Seppi K. (2018). Validation of the MDS clinical diagnostic criteria for Parkinson’s disease. Mov. Disord..

[48] Lohse-Busch H., Marlinghaus E., Reime U., Mowis U. (2014). Focused low-energy extracorporeal shock waves with distally symmetric polyneuropathy (DSPNP): A pilot study. NeuroRehabilitation.

[49] Lohse-Busch H. (2021). Transcranial pulse stimulation (TPS) with focused extracorporeal shock waves. A new promising non invasive symptomatic treatment of Parkinson’s disease. Casuistics Feasibility Study.

[50] Brognara L., Cauli O. (2020). Mechanical Plantar Foot Stimulation in Parkinson’s Disease: A Scoping Review. Diseases.

[51] Martinez-Martin P., Rodriguez-Blazquez C., Alvarez-Sanchez M., Arakaki T., Bergareche-Yarza A., Chade A., Garretto N., Gershanik O., Kurtis M.M., Martinez-Castrillo J.C. (2013). Expanded and independent validation of the Movement Disorder Society-Unified Parkinson’s Disease Rating Scale (MDS-UPDRS). J. Neurol..

[52] van Wamelen D.J., Martinez-Martin P., Weintraub D., Schrag A., Antonini A., Falup-Pecurariu C., Odin P., Ray Chaudhuri K. (2021). The Non-Motor Symptoms Scale in Parkinson’s disease: Validation and use. Acta Neurol. Scand..

[53] Isella V., Mapelli C., Morielli N., Siri C., De Gaspari D., Pezzoli G., Antonini A., Poletti M., Bonuccelli U., Picchi L. (2013). Diagnosis of possible mild cognitive impairment in Parkinson’s disease: Validity of the SCOPA-Cog. Park. Relat. Disord..

[54] Giladi N., Tal J., Azulay T., Rascol O., Brooks D.J., Melamed E., Oertel W., Poewe W.H., Stocchi F., Tolosa E. (2009). Validation of the freezing of gait questionnaire in patients with Parkinson’s disease. Mov. Disord..

[55] Steffen T.M., Hacker T.A., Mollinger L. (2002). Age- and gender-related test performance in community-dwelling elderly people: Six-Minute Walk Test, Berg Balance Scale, Timed Up & Go Test, and gait speeds. Phys. Ther..

[56] Trenkwalder C., Kohnen R., Hogl B., Metta V., Sixel-Doring F., Frauscher B., Hulsmann J., Martinez-Martin P., Chaudhuri K.R. (2011). Parkinson’s disease sleep scale--validation of the revised version PDSS-2. Mov. Disord..

[57] Brown R.G., Dittner A., Findley L., Wessely S.C. (2005). The Parkinson fatigue scale. Park. Relat. Disord..

[58] Williams J.R., Hirsch E.S., Anderson K., Bush A.L., Goldstein S.R., Grill S., Lehmann S., Little J.T., Margolis R.L., Palanci J. (2012). A comparison of nine scales to detect depression in Parkinson disease: Which scale to use?. Neurology.

[59] Stern R.A. (1996). Assessment of Mood States in Neurodegenerative Disease: Methodological Issues and Diagnostic Recommendations. Semin. Clin. Neuropsychiatry.

[60] Rojas-Lechuga M.J., Izquierdo-Dominguez A., Chiesa-Estomba C., Calvo-Henriquez C., Villarreal I.M., Cuesta-Chasco G., Bernal-Sprekelsen M., Mullol J., Alobid I. (2021). Chemosensory dysfunction in COVID-19 out-patients. Eur. Arch. Otorhinolaryngol..

[61] Rosen C.A., Lee A.S., Osborne J., Zullo T., Murry T. (2004). Development and validation of the voice handicap index-10. Laryngoscope.

[62] Zraick R.I., Kempster G.B., Connor N.P., Thibeault S., Klaben B.K., Bursac Z., Thrush C.R., Glaze L.E. (2011). Establishing validity of the Consensus Auditory-Perceptual Evaluation of Voice (CAPE-V). Am. J. Speech Lang. Pathol..

[63] Sauder C., Bretl M., Eadie T. (2017). Predicting Voice Disorder Status from Smoothed Measures of Cepstral Peak Prominence Using Praat and Analysis of Dysphonia in Speech and Voice (ADSV). J. Voice.

[64] Schonenberg A., Prell T. (2022). Measuring quality of life with the Parkinson’s Disease Questionnaire-39 in people with cognitive impairment. PLoS ONE.

[65] Weiner B.J., Lewis C.C., Stanick C., Powell B.J., Dorsey C.N., Clary A.S., Boynton M.H., Halko H. (2017). Psychometric assessment of three newly developed implementation outcome measures. Implement. Sci..

[66] de Climens A.R., Findley A., Bury D.P., Brady K.J.S., Reaney M., Gater A. (2024). Development and Content Validation of the Patient’s Qualitative Assessment of Treatment—Real-World (PQAT-RW): An Instrument to Evaluate Benefits and Disadvantages of Treatments in Real-World Settings. Patient Relat. Outcome Meas..

[67] Yang C., Guo Z., Peng H., Xing G., Chen H., McClure M.A., He B., He L., Du F., Xiong L. (2018). Repetitive transcranial magnetic stimulation therapy for motor recovery in Parkinson’s disease: A Meta-analysis. Brain Behav..

[68] Liu X., Liu H., Liu Z., Rao J., Wang J., Wang P., Gong X., Wen Y. (2021). Transcranial Direct Current Stimulation for Parkinson’s Disease: A Systematic Review and Meta-Analysis. Front. Aging Neurosci..

